# Change in Maxillary Sinus Mucosal Thickness in Patients with Preoperative Maxillary Sinus Mucosal Thickening as Assessed by Otolaryngologists: A Retrospective Study

**DOI:** 10.3390/medicina59101750

**Published:** 2023-09-30

**Authors:** Jin-Hyeong Kim, Eun Jeong Min, Youngkyung Ko, Do Hyun Kim, Jun-Beom Park

**Affiliations:** 1 Department of Periodontics, College of Medicine, The Catholic University of Korea, Seoul 06591, Republic of Korea; jinkjinh@naver.com (J.-H.K.); ko_y@catholic.ac.kr (Y.K.); 2 Department of Medical Life Science, College of Medicine, The Catholic University of Korea, Seoul 06591, Republic of Korea; ej.min@catholic.ac.kr; 3 Department of Biomedicine & Health Sciences, College of Medicine, The Catholic University of Korea, Seoul 06591, Republic of Korea; 4 Dental Implantology, Graduate School of Clinical Dental Science, The Catholic University of Korea, Seoul 06591, Republic of Korea; 5 Department of Otolaryngology-Head and Neck Surgery, Seoul St. Mary’s Hospital, College of Medicine, The Catholic University of Korea, Seoul 06591, Republic of Korea; 6 Department of Medicine, Graduate School, The Catholic University of Korea, Seoul 06591, Republic of Korea

**Keywords:** dental implant, maxillary sinus, sinus floor augmentation, bone transplantation, otolaryngologists

## Abstract

*Background and Objectives:* Maxillary sinus pathologic conditions may increase the risk of complications during posterior maxillary sinus augmentation surgery. The purpose of this study was to evaluate the changes in participants with preoperative maxillary sinus mucosal thickening and to assess this factor as a preoperative risk indicator for sinusitis after maxillary dental implantation. *Materials and Methods:* We compared the preoperative and postoperative maxillary sinus mucosal thickness (MSMT), the distance between the maxillary sinus ostium and sinus floor (MOD), and the MSMT/MOD ratio. The participants were divided into three groups (sinus augmentation, bone grafting, and no grafting). *Results:* The mean preoperative MSMT was 4.3 ± 2.0 mm, and the mean MSMT/MOD ratio was 0.13 ± 0.05. No postoperative sinusitis was observed in these patients, including cases caused by anatomical variations. The mean postoperative MSMT was 4.5 ± 2.3 mm, and the mean postoperative MSMT/MOD ratio was 0.15 ± 0.06. There was no statistically significant difference between the groups at each time point (*p* > 0.05). *Conclusions:* The study found no significant change in MSMT at post-treatment evaluation, even when considering different subgroups. It underscores the importance of preoperative maxillary sinus radiographic assessments and collaboration between dentists and otolaryngologists for better outcomes in patients with preoperative maxillary sinus mucosal thickening.

## 1. Introduction

Dental implants have become increasingly common due to advancements in technology [1,2]. The use of cone-beam computed tomography (CBCT) in dental practice is increasing, and its use is recommended to visualize the anatomy of the maxillary sinus and common pathologies found in the maxilla [3,4]. Maxillary sinus pathologic conditions were evaluated using CBCT, and the frequency of maxillary sinus pathologic entities on CBCT images was 63.5% with mucosal thickening (31.4%), retention cyst (17.1%), partial or complete opacification of the sinus (9.3%), and polypoidal mucosal thickening (5.7%) [5]. There seems to be no consensus yet about the threshold at which thickening of the mucous membrane of the maxillary sinus should be considered pathological, and the definition of maxillary sinusitis varies greatly, even in the scientific literature [6]. A previous study suggested the following classification scheme of maxillary sinus thickness: (1) <2 mm, (2) between 2–5 mm, and (3) >5 mm but confined to the floor of the sinus [7]. A maxillary sinus membrane thickness >2 mm is considered pathologic [8]. In another report, radiographic findings were classified into one of the following five categories based on the type of sinus pathology found: healthy, mucosal thickening >5 mm, multiple mucosal thickening, partial opacity and/or air–fluid levels, and complete opacity [9].

Maxillary sinus augmentation, also known as sinus lift surgery or sinus floor augmentation, is a common dental procedure performed in cases where there is limited bone height or width in the posterior region of the maxilla, specifically in the molar and premolar areas [10]. This procedure is typically carried out to create sufficient bone volume for dental implant placement. During the procedure, the sinus membrane is gently lifted or elevated, creating a space between the sinus floor and the maxilla [11]. Maxillary sinus augmentation can be performed using two primary approaches: the crestal approach and the lateral approach [12]. In the crestal approach, the surgery is conducted through the alveolar ridge where dental implants are to be placed [13]. A small hole is drilled into the bone, and then a series of osteotomes or specialized instruments are used to gently lift the sinus membrane and pack bone graft material into the space created. In the lateral approach, a small window or opening is created in the lateral wall of the maxillary sinus, usually through the lateral wall of the maxilla [14]. This window allows direct access to the sinus membrane, and the bone graft is placed under the membrane to lift the sinus membrane and create space for new bone formation. Bone graft material including autogenous bone, allogenous bone, xenogeneic bone, or synthetic bone is then placed in the created space to promote new bone formation [15]. Placement of dental implants is performed simultaneously or following a healing period of several months, depending on the situations [16].

Maxillary sinus pathologic conditions may increase the risk of complications during posterior maxillary sinus augmentation surgery [5,17,18]. Thickening of the maxillary sinus membrane was reported to be frequently observed on CBCT scans, and it was suggested that this may lead to reductions in the maxillary sinus height [19]. However, chronic sinusitis with thickening of the maxillary sinus membrane does not significantly affect postoperative bone height, healing, or infection scores in patients undergoing sinus augmentation with concurrent implant placement [11]. Similarly, a previous study evaluated the impact of pre-existing maxillary sinus pathology on the survival of dental implants placed at the same time as sinus augmentation and found that the presence of pathology in the maxillary sinus before surgery did not affect the survival rates of dental implants [20]. No significant correlation was found between the frequency and severity of postoperative radiologic changes and the initial condition of the mucosa, sinus anatomy, residual bony height, or type of graft material [21]. A moderate negative correlation was observed between baseline membrane thickness and thickness changes, with thin membranes (<1.56 mm) at baseline becoming thicker by an average of 2.21 [22]. Another report demonstrated that sinus pathology was improved after implant installation [23].

The purpose of this study was to evaluate changes in maxillary sinus mucosal thickness (MSMT) in participants with preoperative maxillary sinus mucosal thickening. The null hypothesis is that there is no significant difference in MSMT between the preoperative and postoperative periods. We analyzed the MSMT, the distance between the maxillary sinus ostium and sinus floor (MOD), and the MSMT/MOD ratio as preoperative risk indicators for sinusitis after maxillary dental implantation.

## 2. Materials and Methods

### 2.1. Study Design

This research protocol was reviewed and approved by the Institutional Review Board of Seoul St Mary’s Hospital, College of Medicine, The Catholic University of Korea (KC23RISI0252, approved 21 April 2023). This is a retrospective study, and a total of nine patients referred to the Department of Otolaryngology between December 2019 and August 2022 were included in this study. Patients were referred from the Department of Periodontology to the Department of Otolaryngology before dental implant installation. All participants underwent CBCT imaging before and after surgery (7.0 ± 1.8 months). Diabetes mellitus, asthma, a past history of endoscopic sinus surgery obstruction of the maxillary sinus ostium, anatomical factors potentially associated with ostial obstruction (such as paradoxical middle turbinate or Haller cells), and the occurrence of postoperative sinusitis were reviewed.

**Figure 1:**
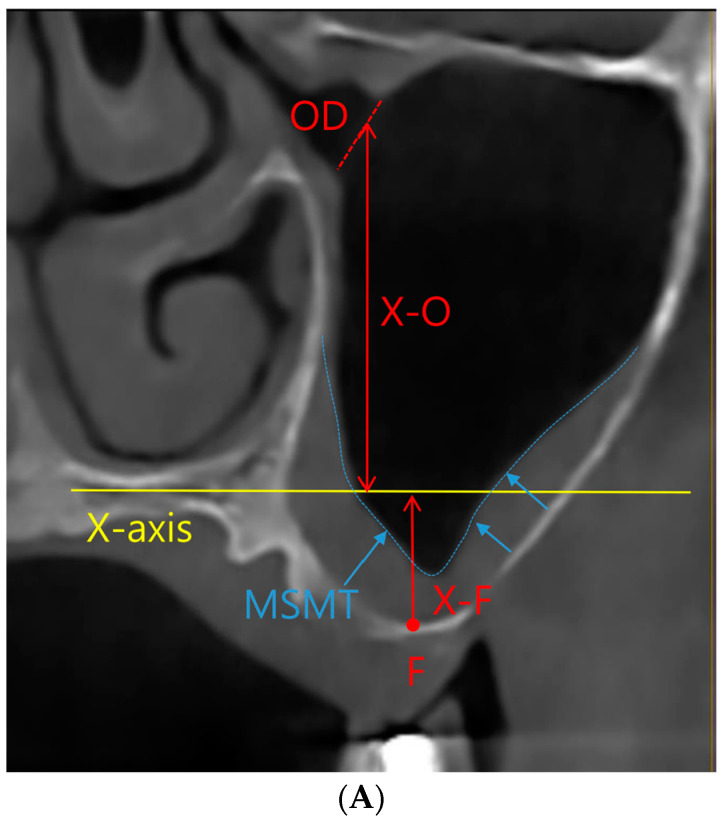

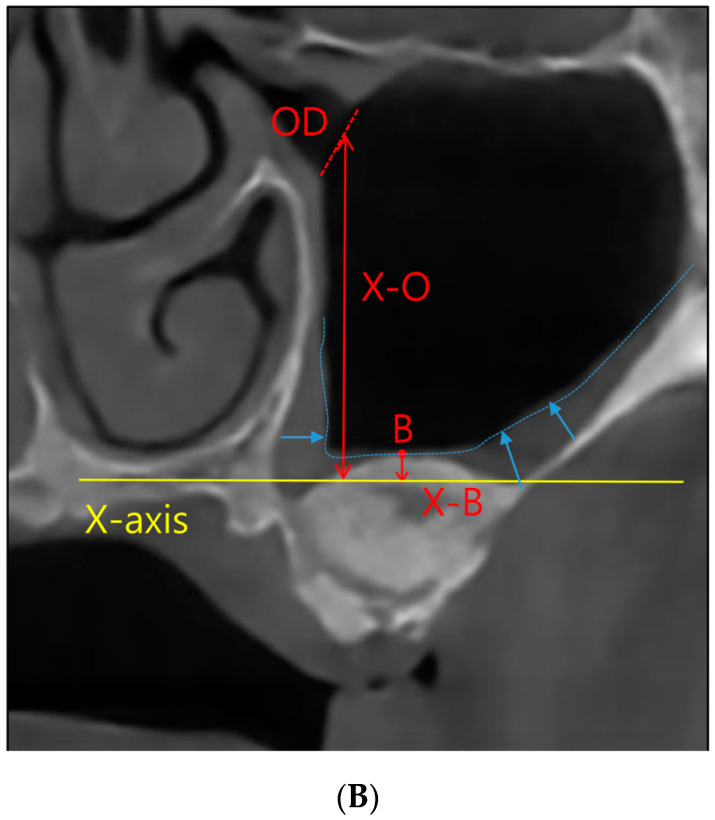
Coronal view of cone-beam computed tomographic images of the maxillary sinus. (**A**) Preoperative view. X-axis, nose floor in coronal view; OD, maxillary sinus ostium diameter; F, the floor of the maxillary sinus; X-F, distance between the X-axis and floor of the maxillary sinus; X-O, distance between the X-axis and maxillary sinus ostium; MSMT, maxillary sinus mucosal thickness; X-F + X-O, maxillary sinus ostium height/distance between the maxillary sinus ostium and sinus floor. (**B**) Postoperative view of sinus augmentation and bone graft. X-B, distance between the X-axis and sinus augmentation, X-F + X-B, increase in alveolar bone height in sinus augmentation.

All study participants underwent CBCT imaging both before and after their surgical interventions, with a mean postoperative imaging interval of 7.0 ± 1.8 months. To provide comprehensive procedural descriptions, InVivoDental (Version 6.0.5, Anatomage, San Jose, CA, USA) was employed for acquiring CBCT images. Subsequently, the images were meticulously exported the Digital Imaging and Communications in Medicine (DICOM) files and meticulously loaded them into InVivo software (Version 6.0.5) for precise measurement. Within this software, we ascertained the preoperative and postoperative values for MSMT, MOD, and the ratio of MSMT to MOD (Figure 1).

**Figure 2:**
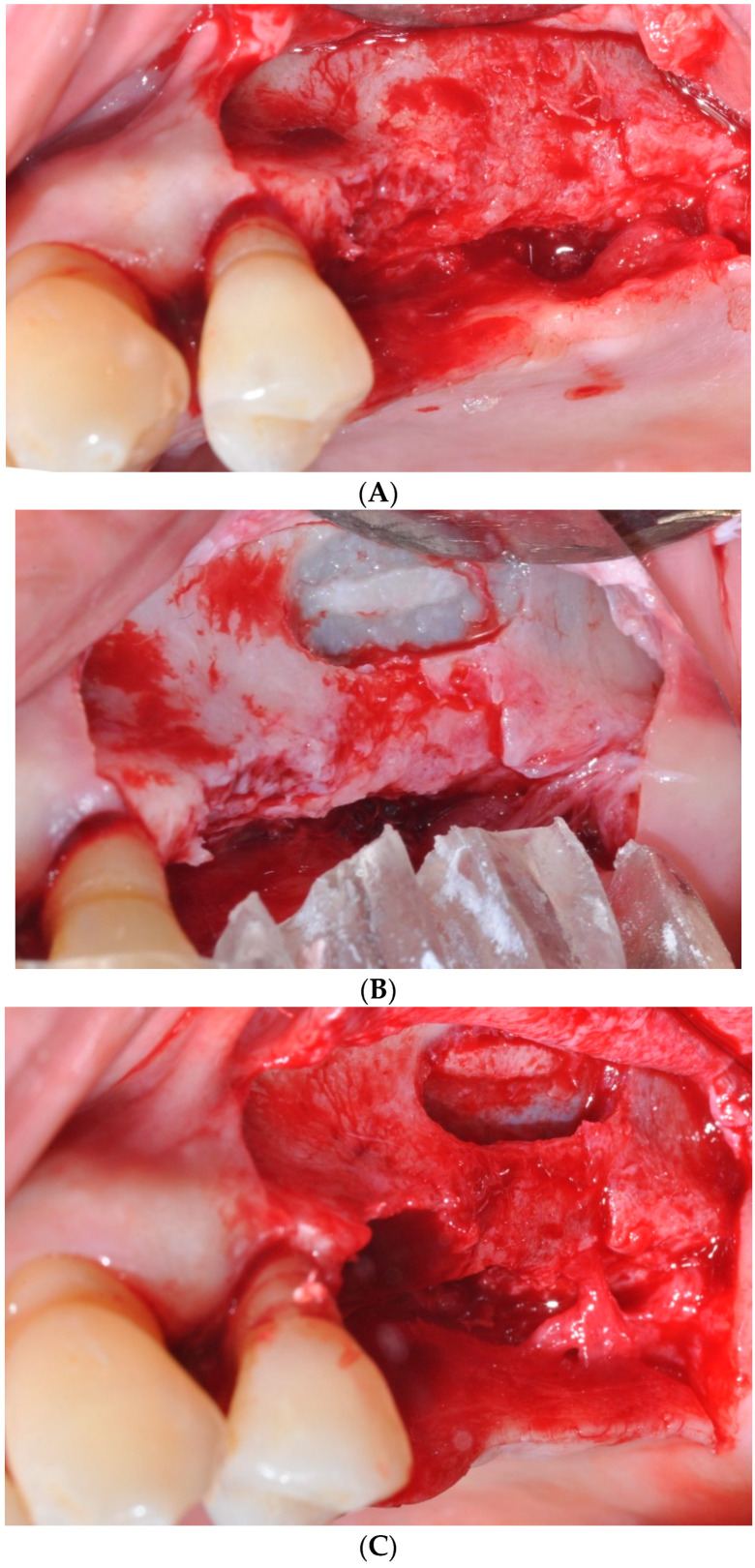

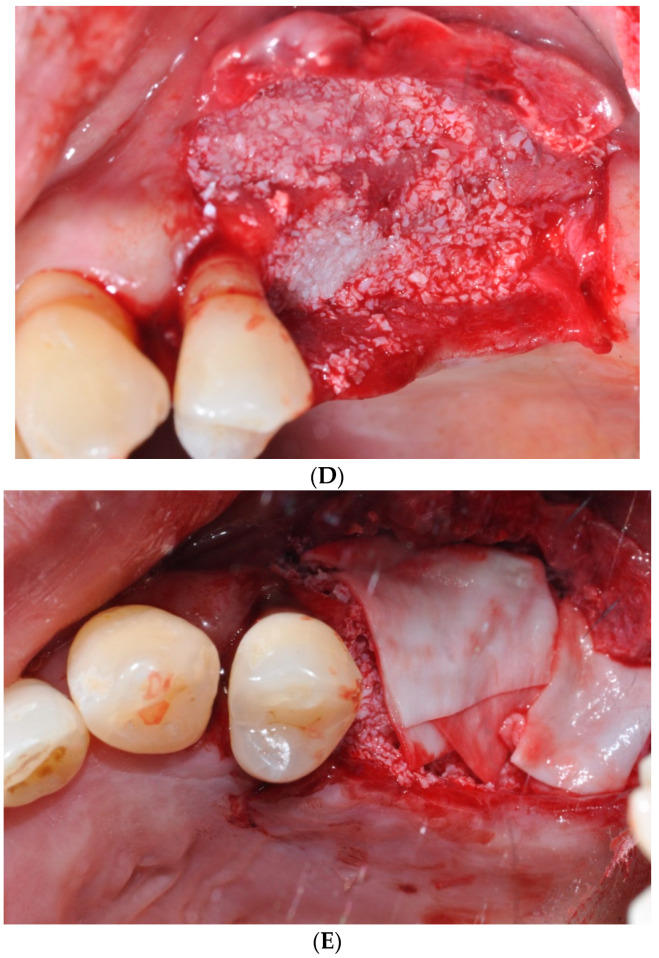
Clinical view of the procedure. (**A**) Buccal view after flap elevation. (**B**) Clinical view at the time of window preparation. (**C**) Buccal view following elevation of the maxillary sinus membrane. (**D**) Sinus augmentation with de-proteinized bovine bone. (**E**) Application of resorbable collagen membrane.

The participants were divided into three groups (sinus augmentation, bone grafting, and no grafting). Three patients each received sinus augmentation, bone grafting, or dental implantation only without sinus augmentation or bone grafting, respectively (Figure 2). The operator carefully elevated the full-thickness flap, gaining access to the surgical site with meticulous precision (Figure 2A). In case of a lateral approach, the low-speed engine with carbide bur with the head size of 0.5–2 mm was used to draw the overall size of the lateral window. Then, the low-speed engine with carbide bur with the head size of 0.5–2 mm was used to make the full cut of the lateral wall (Figure 2B). When the bone cut was fully done, the elevation of the membrane was gently performed with the hand instrument (Figure 2C). This delicate step requires precision and care to avoid any damage to the membrane, ensuring its integrity. The buccal view during this stage showcases the surgeon’s ability to navigate and manipulate the tissue with utmost gentleness, preserving the patient’s sinus health. The sinus membrane along with the bone fragment was elevated, resembling the movement of a trap door, and the void beneath it was then filled with deproteinized bovine bone graft material, specifically Bio-Oss^®^ by Geistlich Pharma in Wolhusen, Switzerland (Figure 2D). Particular attention was given to ensure that the bone graft material was placed adequately on both the mesial and medial sides. Furthermore, extra bone graft material was inserted on both the buccal and palatal sides to expand the width of the ridge. This specialized material is meticulously placed within the sinus cavity to promote new bone growth, providing a stable foundation for future dental implant placement. To protect and stabilize the graft material and facilitate the healing process, a resorbable collagen membrane (BioGide^®^, Geistlich Pharma) is carefully applied over the augmented area (Figure 2E). This membrane acts as a barrier, preventing unwanted tissue intrusion while encouraging natural tissue regeneration. The clinical view at this stage highlights the placement of the membrane, ensuring optimal results and patient comfort during the healing process.

The comprehensive protocols for the bone grafting group are outlined as follows. Before the commencement of the surgical procedure, the patient was instructed to perform a preoperative oral rinse using a 0.12% chlorhexidine mouthwash. Following the successful administration of local anesthesia, a surgical procedure was initiated, involving the reflection of a full-thickness flap. This surgical maneuver facilitated access to and visualization of any buccal or palatal bone dehiscence. The defect site underwent meticulous scrutiny, with particular attention paid to the complete removal of any granulation tissue, ensuring optimal tissue health. Furthermore, both the ridge’s width and the extent of the defect were methodically assessed. The buccal or palatal aspect of the defect site received grafting with deproteinized bovine bone (specifically, Bio-Oss^®^, Geistlich Pharma). A resorbable membrane was carefully contoured and positioned in a saddle-like fashion to effectively cover the bone graft material, promoting optimal graft containment and stability. The surgical site was subsequently closed with sutures, with utmost care taken to minimize flap tension, thereby ensuring the integrity of the surgical closure.

The detailed procedures for the group without grafting (no grafting) are presented as follows. Following the administration of a local anesthetic, consisting of 2% lidocaine with 1:100,000 epinephrine, a precisely designed surgical template was employed to accurately identify the intended implant placement site. A full-thickness flap was carefully elevated, exposing the underlying ridge. The surgical site underwent meticulous preparation using low-speed drills designed for implantation procedures. The dental implant was then judiciously inserted, and when applicable, it was achieved with an insertion torque of 40 Ncm. The closure of the wound was meticulously executed through the use of single sutures, specifically Ethicon^TM^ sutures by Johnson and Johnson MedTech, headquartered in New Brunswick, NJ, USA. Subsequent to implant placement, the surgical site was attentively sutured, with paramount emphasis placed on the minimization of flap tension. This surgical approach was adopted to safeguard the overall integrity of the wound closure, promoting optimal postoperative healing.

### 2.2. Statistic Evaluation

All measured parameters are expressed as mean ± standard deviation values. A normality test was performed, and differences between the groups were analyzed by using the Kruskal–Wallis test, while pre- and post-treatment differences were analyzed by using the Wilcoxon signed-rank test. *p* < 0.05 was considered to indicate statistical significance. All statistical analyses were conducted using Statistical Package for the Social Sciences (SPSS) 12 for Windows (IBM Corporation, Armonk, NY, USA).

## 3. Results

The demographic characteristics of the study participants revealed that the average age of patients was 62.9 years, with a slight variation of ± 7.5 years. Among the enrolled participants, there were six men and three women, constituting the final analysis group. Notably, none of the patients in the study had a medical history of diabetes mellitus or asthma, which could potentially impact their sinus health. It is important to mention that one of the patients had a prior history of endoscopic sinus surgery, a relevant detail in assessing their sinus conditions. Furthermore, the study delved into an in-depth analysis of anatomical variations within the nasal cavity using data from CBCT. This examination identified specific variations, including concha bullosa in two cases, Haller cells in four cases, and a paradoxical curvature of the middle concha in four cases. Importantly, none of the patients exhibited any significant obstruction of the maxillary sinus ostium based on CBCT imaging, suggesting that anatomical variations did not pose an immediate concern. It is noteworthy that the study reported an absence of postoperative sinusitis among the patients, even in cases related to anatomical variations. This suggests that the surgical procedures and treatments employed were effective in preventing such complications.

**Figure 3:**
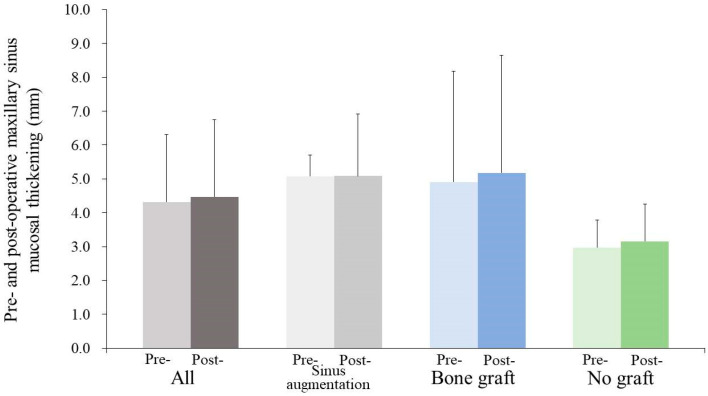
Preoperative and postoperative maxillary sinus mucosal thickness values are presented. There was no statistically significant difference between the groups at each time point (*p* > 0.05).

Regarding the assessment of alveolar bone height increase in sinus augmentation, the study reported an average increase of 6.0 ± 6.0 mm, indicating a positive outcome in terms of bone height augmentation. The study also provided a comprehensive analysis of preoperative MSMT and its relation to the distance between the MOD. The overall mean preoperative MSMT was calculated to be 4.3 ± 2.0 mm (Figure 3). This value was further broken down into specific groups, revealing that in the sinus augmentation group, the mean preoperative MSMT was 5.1 ± 0.6 mm, while in the bone grafting group, it was 4.9 ± 3.3 mm. In the no grafting group, the mean preoperative MSMT was 3.0 ± 0.8 mm. Importantly, statistical analysis indicated that these differences were not statistically significant (*p* > 0.05), suggesting that preoperative MSMT did not significantly vary between these groups.

**Figure 4:**
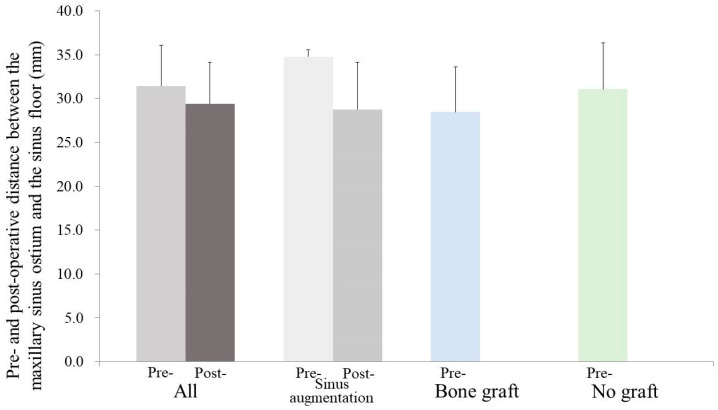
Preoperative and postoperative values of the distance between the maxillary sinus ostium and sinus floor are shown. There was no statistically significant difference between the groups at each time point (*p* > 0.05).

A similar analysis was conducted for the MOD measurements, where the overall mean MOD was reported as 31.4 ± 4.6 mm (Figure 4). Once again, specific group breakdowns were presented, with the sinus augmentation group having a mean MOD of 34.8 ± 0.8 mm, the bone grafting group having a mean MOD of 28.5 ± 5.1 mm, and the no grafting group having a mean MOD of 31.1 ± 5.3 mm. These values did not show statistically significant differences (*p* > 0.05), indicating a lack of significant variation in MOD between the groups.

**Figure 5:**
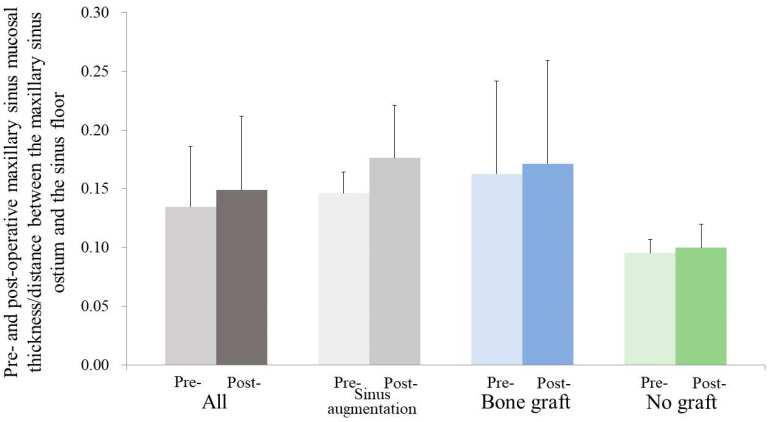
Preoperative and postoperative maxillary sinus mucosal thickness/distance between the maxillary sinus ostium and sinus floor ratios are shown. There was no statistically significant difference between the groups at each time point (*p* > 0.05).

Furthermore, the study assessed the MSMT/MOD ratio, reporting a mean value of 0.13 ± 0.05 (Figure 5). This ratio was then explored within the specific groups, with the sinus augmentation group having a mean MSMT/MOD ratio of 0.15 ± 0.02 mm, the bone grafting group having a mean ratio of 0.16 ± 0.08, and the no grafting group having a mean ratio of 0.10 ± 0.01 mm. Importantly, these group-specific differences were not statistically significant (*p* > 0.05), indicating a lack of significant variation in the MSMT/MOD ratio between the groups.

## 4. Discussion

This comprehensive analysis of patient characteristics, anatomical variations, surgical outcomes, and pre- and postoperative measurements provides a thorough understanding of the study’s findings, highlighting the absence of significant differences in various parameters among the different groups of patients. Among dental factors, the main causes of sinus membrane thickening are periodontitis (47.1%), periapical pathology (23.5%), and root canal treatment (23.1%) [24]. Previous reports have suggested that the severity of the periodontal condition of maxillary molars may influence the degree of maxillary sinus mucosal thickening [25]. In a previous report, researchers analyzed radiographic changes in sinus mucosal thickness in patients with mucosal thickening of odontogenic origin after maxillary molar extraction and sinus augmentation with simultaneous surgical drainage and implant placement at four time points (pre-extraction, preoperatively, immediately after surgery, and post-prosthesis) and determined that sinus mucosal thickness gradually decreased with extraction of the damaged tooth and drainage during sinus augmentation [26]. The risk of postoperative rhinosinusitis has been shown to be greater in patients with chronic sinusitis and when a large amount of graft material was used for sinus surgery [27]. In the current study, variables such as preoperative MSMT, MOD, and MSMT/MOD ratio were used as candidate preoperative risk indicators for maxillary sinusitis.

Following extraction of molars with severe periodontitis, a decrease in thickening of the maxillary sinus membrane was observed regardless of the addition of deproteinized bovine bone mineral to the extraction socket; however, cases with mucosal thickness >2 mm were still frequently observed [28,29]. In the present study, maxillary sinus membrane thickness was compared between the bone graft group and the sinus augmentation group, but no significant differences were found.

Otolaryngologists and dentists see patients suffering from odontogenic maxillary sinusitis on a daily basis [30]. As a result, in surgeries involving the maxillary sinus, both the dentist and otolaryngologist should evaluate the operation and, if necessary, adopt a multidisciplinary approach [31]. A previous report showed that maxillary sinus evaluation using CBCT imaging has led to unsatisfactory agreement between otolaryngologists and oral surgeons [32]. Another study suggested that incidental maxillary sinus imaging findings, such as mucosal thickening, cysts or polyps, and maxillary sinus obstruction, regardless of their severity or size, may not require resolution prior to sinus augmentation and dental implant procedures in asymptomatic patients [33]. Of all the morphologic changes observed, only a small percentage were deemed to require further medical diagnosis and treatment [34]. In a previous report, it was suggested that incidental maxillary sinus imaging findings, such as mucosal edema, cysts, or polyps, regardless of their severity or size and ostial obstruction, may not need to be addressed prior to sinus augmentation and dental implant procedures in asymptomatic patients, but patients with completely opacified sinuses should be referred to an otolaryngologist prior to surgery [33]. While the risk of dental implant-related chronic sinusitis is low in patients with cysts, polyps, or mucosal thickening in the maxillary sinus, prophylactic endoscopic sinus surgery is recommended for patients with intractable chronic sinusitis, fungal sinusitis, and large polyps or cysts [35,36]. In a patient having patent ostium with asymptomatic severe sinus membrane thickening with Haller cells, simultaneous sinus augmentation and implantation resulted in acute sinusitis due to previously undiagnosed fungal colonization [36].

The role of the otolaryngologist in the preoperative and postoperative management of patients with maxillary sinusitis was emphasized in a previous report [37,38]. Nasal endoscopy was suggested to be a better diagnostic tool than traditional panoramic or CBCT [39]. Fiberoptic nasal endoscopy, performed by an otolaryngologist, may allow for a closer look at the middle and lateral nasal wall and can provide evidence of obstruction due to chronic sinusitis or sinus drainage [37]. Clinical evaluation, including nasal endoscopy, is indicated when mucosal thickening and ostiomeatal complex obstruction are identified and surgical correction of ostiomeatal complex obstruction is deemed appropriate to increase success rates and avoid complications that may occur after maxillary sinus augmentation [40].

Depending on the underlying condition causing the thickening of the maxillary sinus membrane, the otolaryngologist may recommend other treatments, such as antibiotics, allergy medication, or surgery [41]. The otolaryngologist may also prescribe antibiotics to treat the infection and stop it from spreading [42]. The microbiome of purulent odontogenic maxillary sinusitis showed anaerobic dominance, and collected bacteria exhibited sufficient susceptibility rates to ampicillin/sulbactam (80%) and piperacillin/tazobactam (93.3%) [30]. The sampled bacteria also displayed sufficient susceptibility to moxifloxacin but poor susceptibility to clindamycin, with only 50% of bacteria being susceptible [30]. Surgery, performed by an otolaryngologist, can remove excess tissue or bone from the sinus cavity to improve drainage and lower the risk of sinusitis [43]. Previously, the Caldwell–Luc method was used to treat chronic sinusitis, but postoperative complications such as buccal skin discomfort and sinusitis recurrence were common following this procedure [44]. For patients with implant-related chronic rhinosinusitis, endoscopic sinus surgery can be used as the first surgical choice with a good prognosis and low morbidity [45].

Depending on the underlying condition causing the thickening of the maxillary sinus membrane, the otolaryngologist may recommend other treatments, such as antibiotics, allergy medication, or surgery [41]. The otolaryngologist may also prescribe antibiotics to treat the infection and stop it from spreading [42]. The microbiome of purulent odontogenic maxillary sinusitis showed anaerobic dominance, and collected bacteria exhibited sufficient susceptibility rates to ampicillin/sulbactam (80%) and piperacillin/tazobactam (93.3%) [30,43]. The sampled bacteria also displayed sufficient susceptibility to moxifloxacin but poor susceptibility to clindamycin, with only 50% of bacteria being susceptible [30]. Surgery, performed by an otolaryngologist, can remove excess tissue or bone from the sinus cavity to improve drainage and lower the risk of sinusitis [44,45]. Previously, the Caldwell–Luc method was used to treat chronic sinusitis, but postoperative complications such as buccal skin discomfort and sinusitis recurrence were common following this procedure [46,47]. For patients with implant-related chronic rhinosinusitis, endoscopic sinus surgery can be used as the first surgical choice with a good prognosis and low morbidity [48].

This current study had several limitations. One of the main limitations is the relatively small sample size used in the study, which may affect the generalizability of the findings to a larger patient population [49]. This present study focused primarily on relatively short-term postoperative assessments. The surgical technique used for maxillary sinus augmentation, including crestal approach and lateral approach, can affect the postoperative outcome [50]. The current design of the study is retrospective in nature, analyzing patient records and clinical and radiographic data to draw conclusions and insights [51]. Prospective studies with larger sample sizes can be considered to validate the findings and further investigate the potential risk factors [52]. Longer-term follow-up will provide a more comprehensive understanding of how maxillary sinus mucosal thickness and sinusitis risk change over time.

## 5. Conclusions

The study found no significant change in MSMT at post-treatment evaluation, even when considering different subgroups. It underscores the importance of preoperative maxillary sinus radiographic assessments and collaboration between dentists and otolaryngologists for better outcomes in patients with preoperative maxillary sinus mucosal thickening.

## Data Availability

All data analyzed during this study are included in this published article.
